# Supplementary material for: Classification of knowledge of fertility period among adolescent girls in East Africa from 2012 to 2022: Machine learning algorithm
Source: PLOS Digit Health. 2026 Feb 23;5(2):e0001108. doi: 10.1371/journal.pdig.0001108 (PMC12928492; doi:10.1371/journal.pdig.0001108)
Supplement: S1 Table — (DOCX) [file pdig.0001108.s001.docx]

| **ML model** | **Performance** | **Unbalanced (%)** | **Balanced (%)** | | | | |
| --- | --- | --- | --- | --- | --- | --- | --- |
|  |  |  | **Under sampling** | **Over sampling** | **SMOTE** | **ADASYN** | **SMOTE-ENN** |
| Decision Tree (DT) | AUC | 62.79 | 62.61 | 86.14 | 85.56 | 85.69 | 95.28 |
|  | Accuracy | 78.25 | 60.78 | 77.86 | 80.71 | 80.45 | 95.42 |
|  | Sensitivity | 24.54 | 59.03 | 84.65 | 83.42 | 82.67 | 96.49 |
|  | Specificity | 90.67 | 62.52 | 71.08 | 78.00 | 78.20 | 94.07 |
|  | PPV | 37.84 | 61.15 | 74.53 | 79.13 | 79.38 | 95.38 |
|  | NPV | 83.86 | 60.43 | 82.24 | 82.47 | 81.63 | 95.48 |
| Random Forest (RF) | AUC | 68.44 | 68.69 | 89.54 | **91.12*** | 91.08 | 99.74 |
|  | Accuracy | 81.05 | 62.48 | 80.16 | **83.26*** | 82.86 | 97.80 |
|  | Sensitivity | 21.34 | 63.61 | 85.01 | **85.44*** | 85.40 | 98.01 |
|  | Specificity | 94.86 | 61.35 | 74.32 | **81.07*** | 79.97 | 97.53 |
|  | PPV | 49.02 | 62.19 | 77.01 | **81.87*** | 81.29 | 98.05 |
|  | NPV | 83.90 | 62.78 | 84.16 | **84.77*** | 84.46 | 97.47 |
| K-Nearest Neighbor  (KNN) | AUC | 64.71 | 66.99 | 76.44 | 86.79 | 85.81 | 98.27 |
|  | Accuracy | 79.82 | 61.60 | 67.85 | 78.79 | 77.81 | 94.52 |
|  | Sensitivity | 20.02 | 63.02 | 56.36 | 80.59 | 78.71 | 95.61 |
|  | Specificity | 93.66 | 60.17 | 79.35 | 76.99 | 76.90 | 93.13 |
|  | PPV | 42.21 | 61.26 | 73.18 | 77.79 | 77.58 | 94.64 |
|  | NPV | 83.50 | 61.95 | 64.52 | 79.86 | 78.05 | 94.35 |
| Logistic Regression  (LR) | AUC | **74.38*** | 72.22 | 73.24 | 89.91 | 89.99 | 97.09 |
|  | Accuracy | **82.71*** | 65.52 | 66.13 | 81.35 | 81.47 | 91.77 |
|  | Sensitivity | **18.91*** | 64.79 | 62.69 | 74.37 | 74.06 | 90.76 |
|  | Specificity | **97.47*** | 66.25 | 69.56 | 88.33 | 88.99 | 93.04 |
|  | PPV | **63.38*** | 65.74 | 67.32 | 86.43 | 87.23 | 94.30 |
|  | NPV | **83.86*** | 65.31 | 65.09 | 77.51 | 77.16 | 88.82 |
| Artificial Neural Network  (ANN) | AUC | 71.12 | 67.67 | 80.59 | 90.79 | 90.83 | 98.67 |
|  | Accuracy | 81.12 | 61.60 | 72.44 | 82.01 | 82.18 | 96.00 |
|  | Sensitivity | 22.71 | 62.63 | 77.83 | 79.59 | 81.66 | 95.87 |
|  | Specificity | 94.64 | 60.56 | 67.05 | 84.44 | 82.71 | 96.17 |
|  | PPV | 49.50 | 61.35 | 70.26 | 83.64 | 82.75 | 96.95 |
|  | NPV | 84.11 | 61.86 | 75.15 | 80.53 | 81.62 | 94.83 |
| Naïve Bayes  (NB) | AUC | 72.27 | 69.79 | 71.17 | 75.85 | 74.14 | 87.61 |
|  | Accuracy | 64.64 | 63.33 | 64.55 | 67.86 | 66.01 | 79.84 |
|  | Sensitivity | 66.43 | 76.31 | 74.71 | 82.20 | 85.24 | 82.05 |
|  | Specificity | 64.22 | 50.36 | 54.39 | 53.52 | 46.48 | 77.05 |
|  | PPV | 30.05 | 60.57 | 62.09 | 63.88 | 61.80 | 81.93 |
|  | NPV | 89.21 | 68.02 | 68.26 | 75.04 | 75.62 | 77.19 |
| Support Vector Machine  (SVM) | AUC | 67.97 | 70.73 | 78.40 | 90.30 | 90.30 | 98.66 |
|  | Accuracy | 82.85 | 65.03 | 71.12 | 82.72 | 82.74 | 94.66 |
|  | Sensitivity | 19.90 | 64.40 | 73.99 | 76.65 | 79.71 | 93.37 |
|  | Specificity | 97.41 | 65.66 | 68.26 | 88.78 | 85.83 | 96.33 |
|  | PPV | 64.00 | 65.21 | 69.98 | 87.23 | 85.10 | 96.99 |
|  | NPV | 84.02 | 64.86 | 72.41 | 79.18 | 80.64 | 91.98 |
| Extreme Gradient Boost  (XGB) | AUC | 73.18 | 70.41 | 80.03 | 90.92 | 90.89 | 98.63 |
|  | Accuracy | 82.58 | 63.66 | 71.28 | 82.53 | 82.45 | 95.11 |
|  | Sensitivity | 20.62 | 65.05 | 72.56 | 79.79 | 81.81 | 94.49 |
|  | Specificity | 96.91 | 62.26 | 69.99 | 85.27 | 83.10 | 95.90 |
|  | PPV | 60.69 | 63.27 | 70.74 | 84.41 | 83.09 | 96.70 |
|  | NPV | 84.07 | 64.06 | 71.84 | 80.84 | 81.82 | 93.21 |
| Adaptive Boost  (ADB) | AUC | 73.63 | 71.12 | 65.23 | 74.51 | 72.78 | 86.55 |
|  | Accuracy | 82.39 | 63.53 | 72.16 | 66.99 | 66.77 | 78.09 |
|  | Sensitivity | 12.04 | 65.45 | 64.23 | 68.36 | 74.64 | 84.99 |
|  | Specificity | 98.67 | 61.61 | 66.23 | 65.62 | 58.79 | 69.34 |
|  | PPV | 67.65 | 63.01 | 65.54 | 66.53 | 64.78 | 77.86 |
|  | NPV | 82.90 | 64.08 | 64.93 | 67.47 | 69.54 | 78.46 |
| Cat Boost  (CatB) | AUC | 73.59 | 71.11 | 80.25 | 91.04 | 90.95 | 98.55 |
|  | Accuracy | 82.85 | 64.48 | 71.40 | 82.77 | 82.60 | 95.25 |
|  | Sensitivity | 20.29 | 66.43 | 72.56 | 78.52 | 79.68 | 94.44 |
|  | Specificity | 97.32 | 62.52 | 70.23 | 87.03 | 85.57 | 96.27 |
|  | PPV | 63.66 | 63.92 | 70.91 | 85.82 | 84.87 | 96.98 |
|  | NPV | 84.07 | 65.08 | 71.91 | 80.20 | 80.57 | 93.18 |
| **Note:** The best performance of machine learning algorithm was represented by * | | | | | | | |
