# Supplementary material for: Classification of knowledge of fertility period among adolescent girls in East Africa from 2012 to 2022: Machine learning algorithm
Source: PLOS Digit Health. 2026 Feb 23;5(2):e0001108. doi: 10.1371/journal.pdig.0001108 (PMC12928492; doi:10.1371/journal.pdig.0001108)
Supplement: Attachment — Submitted filename: Response to Reviewers.docx [file pdig.0001108.s002.docx]

**Point by point responses**

**Author summery**

The purpose of this study was to classify knowledge of the fertility period among adolescent girls in East Africa. Ten machine-learning models were trained, and the random forest model provided the most accurate classification with an AUC score of 91.12% and an accuracy of 83.26%.  Using the SHAP feature importance method, the top ten predictors were identified: education level, country, heard about family planning, heard about sexually transmitted infections, wealth index, knowledge of any method, and visited health facilities. Education level was a primary factor since girls with higher education levels were better prepared to understand reproductive health concepts. Similarly, increased exposure to hearing about family planning and general knowledge about STIs improved knowledge of fertility, again showing that a more extensive health communication strategy has contributed to girls' foundational knowledge. It is important to note that the wealth index also has an effect on access to media, education, and health services. We saw that girls who demonstrated that they were aware of a contraceptive method or had ever visited a health facility were significantly more likely to understand their fertility period.

1. **Scientific Content and Justification**

- **You have applied machine learning (ML) to classify knowledge of the fertility period (KFP). However, you have not clearly explained why ML is needed over traditional statistical methods.**

Consequently, as the number of input variables and potential correlations increases, traditional statistical methods have shown limitations in handling large and complex datasets [1]. Machine learning offers a practical solution to these challenges by effectively capturing intricate and nonlinear relationships within the data and supporting more robust, data-driven decision-making compared to earlier statistical approaches [2, 3]. Statistical analysis methods do not uncover hidden information and cannot identify predictive patterns and rules to inform evidence-based decision-making [4]. Furthermore, many studies currently recommend artificial intelligence, data mining, and machine learning algorithms as ways to predict healthcare service delivery, such as the use of modern family planning, with different machine learning algorithms [5]. Machine learning algorithms help predict problems and their determinants using large data sets that assist public health decision-making research [6].

- **Please explain how ML improves prediction or policy insights in this context. You may consider comparing with logistic regression outputs as a baseline.**

Machine learning (ML) offers distinct advantages over traditional statistical methods like logistic regression when classifying knowledge of the fertility period (KFP) among adolescent girls. While logistic regression is effective for modeling linear relationships and interpreting variable significance, it assumes independence among predictors and may struggle with complex, nonlinear interactions [4]. In contrast, ML algorithms such as random forest, support vector machines, and boosting techniques can capture intricate patterns and interactions between variables without requiring strict assumptions. This flexibility allows ML models to uncover hidden relationships in large, multidimensional datasets, improving predictive accuracy and robustness [1].

The use of machine learning enabled the identification of key determinants and the development of predictive models that classify fertility period knowledge more effectively. This classification is vital for bridging gaps in awareness and beliefs, which in turn supports efforts to reduce financial barriers, maternal and child mortality, and societal pressures. The findings offer actionable guidance for policymakers and program managers, suggesting targeted interventions such as promoting awareness of family planning and sexually transmitted infections, strengthening health education, expanding access to quality health services, and refining health education policy frameworks.

2. **Dependent variables and operational definition**

The dependent variable of this study was the knowledge of fertility periods, which was classified into two categories: good knowledge of fertility periods and poor knowledge of fertility periods [7, 8].

**Good knowledge of the fertility period:** Knowledge of the fertility period was assessed by one standardized question: "When do you think a woman's fertility period is?" Respondents that answered "In the middle of the menstrual cycle" were classified as having good knowledge about the fertility period [7, 9-11].

**Poor knowledge of the fertility period:** The same question, "When do you think a woman's fertility window is?" was used to assess poor awareness. Any respondent that answered "During her period," "After her period finished," or "Before her period started, at any time/course of a month," or "We don't know" was categorized as having poor knowledge about fertility periods [7, 8, 10, 11].

3. **Methodology and Model Transparency**

- **Data preprocessing**

Data preprocessing refers to the process of transforming raw, messy data to a clean, proper format that can be used for analysis, machine learning, or similar data-driven processes [12]. Data cleaning, class balancing, feature selection, and data splitting were among the data preparation techniques used in this study. These processes guarantee the data can be consistent, accurate, and suitable for modeling, which improves analytical algorithms' performance and reliability. Research shows that effective preprocessing can increase the accuracy of a model and reduce computational costs, making it a crucial step in any data-driven workflow [13].

- **Handling of missing data**

Data cleaning was applied to address noise and missing values. It is impossible to send raw data through a training process and model testing since there might be missing data and the possibility of biased or misinformed results [14]. This dataset had no missing values, and missing value imputation techniques were not applied.

- **You have used 10 ML algorithms and SMOTE for class balancing. While this is good, the reason for choosing SMOTE over other techniques is not clearly explained.**

When models are trained on imbalanced datasets, their performance generally gives priority to the majority class, leading to predictions that are biased and the minority-class instances being usually misclassified [15]. To address these issues, under-sampling, over-sampling, Synthetic Minority Over-sampling Technique (SMOTE), Adaptive Synthetic Sampling (ADASYN), and Synthetic Minority Over-sampling Technique Edited Nearest Neighbor (SMOTE-ENN) techniques were assessed [16]. Out of the tested resampling methods, under-sampling had the lowest performance and was omitted due to there being under fitting. Although oversampling and the ADASYN technique performed better than under sampling, their results were still much lower than those of SMOTE and SMOTE-ENN. Although SMOTE-ENN had the highest performance, it was eventually dropped because of overfitting. Thus, SMOTE was selected as the best method for balancing the training dataset relating to knowledge of the fertility period among adolescent girls.

- **Feature selection steps (Boruta output)**

Feature selection is a method of dimension reduction, which entailed determining which independent variables were most pertinent and had the biggest influence on the target variable's classification [17]. In this study, feature selection was done using the Boruta algorithm. The Boruta algorithm determined which features in the dataset were highly and weakly relevant for the outcome variable. To select the features using the Boruta algorithm, we followed different steps, such as installing and loading required packages, preparing the dataset, running the Boruta algorithm, checking and plotting results, resolving tentative features, and extracting selected features.

- **Hyperparameter tuning: why did default settings perform better than grid search or Optuna?**

In this study, the default setting performed better than the grid search or Optuna. Because random forest is known for its low sensitivity to hyperparameters, especially in a balanced dataset [18]. Random forests typically allow trees to grow deep, which helps capture complex interactions. Tuning often restricts depth or splits to avoid overfitting, but in ensemble models, this can reduce diversity and weaken predictive [19].

4. **Performance metrics**

- **You have reported accuracy and AUC but not F1-score, confusion matrix, or precision-recall. These are important, especially with imbalanced data.**

When it comes to understanding adolescents' knowledge of fertility periods, specifically regarding imbalanced data, sensitivity, specificity, PPV, NPV, accuracy, and AUC are more appropriate than F1-score, precision, and recall [20]. These metrics are more closely aligned to evaluate aspects of public health and clinical decision-making. For example, sensitivity and specificity can be used to examine the rate at which the model identifies adolescents with good versus poor knowledge of the fertility period.  Because PPV and NPV account for prevalence, PPV and NPV are therefore more actionable on a population level in terms of next steps for educational interventions. AUC and accuracy also provide evaluators some information that is not contingent on the location of a threshold [21]. These metrics have been widely used in reproductive health and other clinical studies for virtually the same reasons as to why they are most suited to altruism and public health methods. F1-score and precision-recall are often seen as threshold-aware and less intuitive for stakeholders in the field of health education [22].

- **Please include confidence intervals for AUC and other metrics.**

In this study, confidence intervals were not used, because in many machine learning applications, especially those focused on model development, feature selection, or comparative performance, confidence intervals for AUC and other metrics are not strictly required [23]. AUC is often used as a point estimate to rank models or assess general discrimination ability, and the emphasis is placed on cross-validation performance rather than statistical inference.

5. **Interpretation of SHAP and findings**

- **SHAP plots are a strength, but their interpretation is not clear. Explain what the plots mean in simple terms and relate them to the real-world context.**

### **Global feature importance**

SHAP global feature importance was applied using a random forest classifier to identify the most influential predictors of adolescent girls’ knowledge of the fertility period. Each independent variable was evaluated based on its mean absolute SHAP value (MASHAPV), which measures the average contribution of that variable to the model’s predictions. Variables were ranked in descending order of importance, with higher MASHAPV indicating greater influence. The top predictors included having a secondary education level and residing in Mozambique or Madagascar, which emerged as the strongest determinants of knowledge of fertility period. Additional important features included hearing about family planning, living in Rwanda, hearing about sexually transmitted infections, belonging to the rich wealth index, knowledge of any contraceptive method, visiting health facilities, and residing in Comoros were determinants of knowledge of fertility period, as illustrated in Figure 9.

### **Model interpretation**

The beeswarm plot provides a compact visual summary of how each feature influences the model’s prediction of whether adolescent girls have knowledge of their fertility period.  Each dot represents an individual prediction, with color indicating the feature value (i.e., blue for low value and red for high value) and positions on the X-axis showing the direction and strength of impact. Dots on the right side (positive SHAP values) increase the likelihood of knowledge of the fertility period, while those on the left (negative SHAP values) decrease it.

In this model several features strongly increased the probability of knowledge of the fertility period; in Figure 10, adolescent girls who had secondary education, were from Madagascar or Comoros, had heard about family planning or sexually transmitted infections, were in the rich wealth index category, had knowledge of any contraceptive method, and had visited health facilities. These factors reflect greater access to education, health services, and reproductive information. Conversely, being from Mozambique or Rwanda was associated with a decreased likelihood of knowledge of the fertility period, reflecting regional disparities in health education outreach or access. The beeswarm plot thus highlights both individual-level and country-level influences, offering actionable insights for targeted interventions in adolescent reproductive health programs.

### **Local feature importance with SHAP**

The SHAP waterfall plot in Figure 11  explains how individual features contributed to the model’s prediction for one adolescent girl regarding her knowledge of the fertility period. The plot begins at the average model output (E[f(x)] = -1.577), which represents the baseline prediction before considering any personal characteristics. Each feature then pushes the prediction upward (red bars) or downward (blue bars) depending on whether it increases or decreases the likelihood of knowledge of the fertility period.

In this specific case, the final model output was f(x) = -1.873, meaning the model predicted that the adolescent girl did not have knowledge of her fertility period. This outcome was shaped by several negative influences: she had no media exposure, was not in the rich wealth category, had not heard about family planning, and was from Ethiopia but not Madagascar, all of which pulled the prediction downward. However, some features pushed the prediction upward: she had secondary education, had visited health facilities, was not from Mozambique, and had knowledge of contraceptive methods, indicating partial access to reproductive health resources.

- **You list top features (e.g., education, wealth, country) but do not explain how these insights can help program planners or health educators.**

By using the random forest classifier algorithm with SHAP, the top ten determinant factors of knowledge of the fertility period among adolescent girls were identified. Those determinant factors were education level, country, hearing about family planning, hearing about sexually transmitted infections, wealth index, knowledge of any method, and visiting health facilities. These insights provide important information to program planners and health educators to help plan interventions that are targeted, culturally relevant, and effective. With an understanding of who is informed, who had no access, and where the gaps are, program planners and health educators can better tailor messaging, allocate resources, and refine outreach to foster healthier behaviors and equitable access to care.

6. **Language and grammar**

The issue of language and grammar already solved.

7. **Ethics and data availability**

We are trying to share and upload the code on GitHub.

8. **Conclusion and implication**

- **Your conclusion repeats the results but does not offer clear recommendations for policy, programs, or further research.**

This study used machine-learning algorithms to identify key factors influencing adolescent girls’ knowledge of the fertility period in East Africa, revealing a low awareness rate of just 13.22%. Among ten models tested, the random forest classifier performed best on balanced data, identifying top predictors such as education level, country of residence, exposure to family planning and STI information, wealth index, knowledge of contraceptive methods, and visits to health facilities. These findings highlight significant disparities in reproductive health knowledge across socioeconomic and geographic lines, emphasizing the need for targeted, data-driven interventions.

Based on these insights, governments and NGOs should prioritize integrating comprehensive sexuality education into secondary school curricula, especially in countries with lower awareness levels like Mozambique and Rwanda. Programs should also expand youth-friendly services, enhance outreach through media and community platforms, and subsidize reproductive health services for low-income adolescents. Further research is needed to explore the impact of STI education on fertility awareness and to understand barriers to accessing health services. These actions can help bridge knowledge gaps, promote informed decision-making, and improve reproductive health outcomes among adolescent girls in the region.

- **Please state how governments or NGOs can use your findings, especially in adolescent reproductive health programs.**

Governments, NGOs, policy makers, and researchers can utilize these findings to design targeted interventions for improving adolescents’ reproductive health based on the identified gaps and disparities. Education level could inform the development of age-appropriate, literacy-sensitive materials. Country-specific data will ensure culturally and policy-sensitive programming. If many adolescents report they have not heard about either family planning or STIs, then expanded outreach efforts through schools and the media, as well as peer education, would be warranted. The wealth index would assist in framing free or subsidized services and adopting a focus on low-income communities. Understanding knowledge of any method would also guide programs to reduce misinformation and promote a wider range of contraceptive options. Last, data on whether adolescents have ever visited health facilities would provide invaluable information on making youth-friendly services adequately available for care, reducing stigma around care, and making accessibility to care a priority. These findings also promote future research into exploring the effectiveness of current communication channels or barriers to accessing service among marginalized youth.

**References**

1.	Chen, J.H. and S.M. Asch, Machine Learning and Prediction in Medicine - Beyond the Peak of Inflated Expectations. N Engl J Med, 2017. **376**(26): p. 2507-2509.

2.	Ij, H., Statistics versus machine learning. Nat Methods, 2018. **15**(4): p. 233.

3.	Mukthar, V.K., et al., The uptake of modern contraceptives among women of reproductive age attending maternal child health and family planning (MCH/FP) clinics. East African Medical Journal, 2014. **91**(12): p. 457-466.

4.	Yehuala, T.Z., Exploring machine learning algorithms to predict not using modern family planning methods among reproductive age women in East Africa. BMC Health Services Research, 2024. **24**(1): p. 1595.

5.	Ngiam, K.Y. and W. Khor, Big data and machine learning algorithms for health-care delivery. The Lancet Oncology, 2019. **20**(5): p. e262-e273.

6.	López-Martínez, F., et al., A case study for a big data and machine learning platform to improve medical decision support in population health management. Algorithms, 2020. **13**(4): p. 102.

7.	Getahun, M.B. and A.G. Nigatu, Knowledge of the ovulatory period and associated factors among reproductive women in Ethiopia: a population-based study using the 2016 Ethiopian Demographic Health Survey. International journal of women's health, 2020: p. 701-707.

8.	Fente, B.M., et al., Knowledge of fertility period among reproductive age women in Kenya: a multilevel analysis based on 2022 Kenyan demographic health survey. Contraception and Reproductive Medicine, 2024. **9**(1): p. 27.

9.	Wolde, M., et al., Knowledge of Fertile period and its determinants among women of childbearing age in Ethiopia: a multilevel analysis based on 2016 Ethiopian Demographic and Health Survey. Frontiers in Public Health, 2022. **10**: p. 828967.

10.	Sundaram, A., et al., Contraceptive failure in the United States: estimates from the 2006–2010 National Survey of Family Growth. Perspectives on sexual and reproductive health, 2017. **49**(1): p. 7-16.

11.	Morris, J.L. and H. Rushwan, Adolescent sexual and reproductive health: The global challenges. International Journal of Gynecology & Obstetrics, 2015. **131**: p. S40-S42.

12.	Yasodha, D., Data Preprocessing Methods for Machine Learning: Empirical Comparison. International Journal for Multidisciplinary Research, 2025.

13.	Koukaras, P. and C. Tjortjis, Data Preprocessing and Feature Engineering for Data Mining: Techniques, Tools, and Best Practices. AI, 2025. **6**(10): p. 257.

14.	Emmanuel, T., et al., A survey on missing data in machine learning. Journal of Big data, 2021. **8**(1): p. 140.

15.	Luque, A., et al., The impact of class imbalance in classification performance metrics based on the binary confusion matrix. Pattern Recognition, 2019. **91**: p. 216-231.

16.	Setiawan, B.D., U. Serdült, and V. Kryssanov, A machine learning framework for balancing training sets of sensor sequential data streams. Sensors, 2021. **21**(20): p. 6892.

17.	Brownlee, J., Data preparation for machine learning: data cleaning, feature selection, and data transforms in Python. 2020: Machine Learning Mastery.

18.	Probst, P., M.N. Wright, and A.L. Boulesteix, Hyperparameters and tuning strategies for random forest. Wiley Interdisciplinary Reviews: data mining and knowledge discovery, 2019. **9**(3): p. e1301.

19.	Fernández-Delgado, M., et al., Do we need hundreds of classifiers to solve real world classification problems? The journal of machine learning research, 2014. **15**(1): p. 3133-3181.

20.	Fernández, A., et al., SMOTE for learning from imbalanced data: progress and challenges, marking the 15-year anniversary. Journal of artificial intelligence research, 2018. **61**: p. 863-905.

21.	Wong, H.B. and G.H. Lim, Measures of diagnostic accuracy: sensitivity, specificity, PPV and NPV. Proceedings of Singapore healthcare, 2011. **20**(4): p. 316-318.

22.	Brownlee, J., Imbalanced classification with Python: better metrics, balance skewed classes, cost-sensitive learning. 2020: Machine Learning Mastery.

23.	Saito, T. and M. Rehmsmeier, The precision-recall plot is more informative than the ROC plot when evaluating binary classifiers on imbalanced datasets. PloS one, 2015. **10**(3): p. e0118432.
