# Supplementary material for: Classification of knowledge of fertility period among adolescent girls in East Africa from 2012 to 2022: Machine learning algorithm
Source: PLOS Digit Health. 2026 Feb 23;5(2):e0001108. doi: 10.1371/journal.pdig.0001108 (PMC12928492; doi:10.1371/journal.pdig.0001108)
Supplement: Attachment — Submitted filename: Response to Reviewer.docx [file pdig.0001108.s003.docx]

**Date: Nov** 10, 2025

**Dear editorial board member (s) of** **PLOS Digital Health** **journal**

We the authors have been requested to revise the manuscript entitled ***“Classification of knowledge of fertility period among adolescent girls in East Africa from 2012 to 2022: Machine learning algorithm”,*** and identified by Manuscript ID PDIG-D-25-00447R1 under **PLOS Digital Health** journal, which was submitted for publication. So, we received the editor’s and reviewer’s revision comments for the betterment of the manuscript before its publication.

We are very thankful for receiving comments, suggestions, directions, questions, and deep insights from the editor and reviewers. We are very happy in receiving constructive and valuable comments for the betterment of the manuscript. Accordingly, we have considered all the comments, questions, directions, and suggestions and provided a point-by-point response letter.

Finally, we have submitted all the required documents in their revised form. We hope that we have addressed all the suggestions, directions, and raised questions and if you believe that point(s) is not addressed, please let us know.

**Thank you very much all editor (s), and reviewers**

**On behalf of the authors**

**Yours sincerely,**

**Andualem Addisu Birlie (Correspondence author)**

**Point-by-point response letter**

**Editor comments**

PDIG-D-25-00447R1

Classification of knowledge of fertility period among adolescent girls in East Africa from 2012 to 2022: Machine learning algorithm.

PLOS Digital Health

Dear Dr. Birlie,

Thank you for submitting your manuscript to PLOS Digital Health. After careful consideration, we feel that it has merit but does not fully meet PLOS Digital Health's publication criteria as it currently stands. Therefore, we invite you to submit a revised version of the manuscript that addresses the points raised during the review process.

Please submit your revised manuscript by Jan 04 2026 11:59PM. If you will need more time than this to complete your revisions, please reply to this message or contact the journal office at [digitalhealth@plos.org](mailto:digitalhealth@plos.org#inbox/_blank). When you're ready to submit your revision, log on to [https://www.editorialmanager.com/pdig/](https://www.editorialmanager.com/pdig/#inbox/_blank) and select the 'Submissions Needing Revision' folder to locate your manuscript file.

Please include the following items when submitting your revised manuscript:

* A rebuttal letter that responds to each point raised by the editor and reviewer(s). You should upload this letter as a separate file labeled 'Response to Reviewers'. This file does not need to include responses to any formatting updates and technical items listed in the 'Journal Requirements' section below.

* A marked-up copy of your manuscript that highlights changes made to the original version. You should upload this as a separate file labeled 'Revised Manuscript with Track Changes'.

* An unmarked version of your revised paper without tracked changes. You should upload this as a separate file labeled 'Manuscript'.

If you would like to make changes to your financial disclosure, competing interests statement, or data availability statement, please make these updates within the submission form at the time of resubmission. Guidelines for resubmitting your figure files are available below the reviewer comments at the end of this letter.

We look forward to receiving your revised manuscript.

Kind regards,

Laura Sbaffi, PhD, MA, MSc

Section Editor

PLOS Digital Health

Laura Sbaffi

Section Editor

PLOS Digital Health

Leo Anthony Celi

Editor-in-Chief

PLOS Digital Health

[orcid.org/0000-0001-6712-6626](http://orcid.org/0000-0001-6712-6626#inbox/_blank)

**Authors Response:** Dear Laura Sbaffi, PhD, MA, MSc (Section Editor, PLOS Digital Health), Laura Sbaffi (Section Editor, PLOS Digital Health) and Leo Anthony Celi (Editor-in-Chief, PLOS Digital Health), we thank you for recommending us read and respond to all the peer reviewer comments and requesting us to revise our work for its betterment. We hope that we have addressed all the mentioned comments, questions, and suggestions that were raised by the reviewers. All the required documents (revised manuscript, tracked manuscript, and point-by-point response) also have been submitted. If there is any issue that is not addressed, please let us know!!

Thank you so much!!

**Editorial Comments to Author**

**Thank you in advance. We have prepared the point by point response for related questions/comments. We appreciate your attention to these details and hope these revisions enhance the clarity and quality of the manuscript.**

Please provide an Author Summary. This should appear in your manuscript between the Abstract (if applicable) and the Introduction, and should be 150–200 words long. The aim should be to make your findings accessible to a wide audience that includes both scientists and non-scientists.

**Author response:** Thank you for your guidance. The Author Summary has been added to the manuscript in the appropriate place, located between the Abstract and the Introduction. The Author Summary has been written adhering to the word limits: 150-200 words, and we have written it for a general audience including scientist and non-scientist readers. If any additional edits are required, please do not hesitate to let us know.

**Reviewer comments to Author**

**Reviewer #1:**

Thank you for your submission. Your study addresses an important topic in adolescent reproductive health using modern machine learning methods. The use of DHS datasets from 12 East African countries adds strength to your analysis. However, the manuscript needs major revision to improve scientific clarity, transparency, and presentation. Below are my detailed comments:

**Author response:** We are grateful for your thoughtful and constructive feedback. Your acknowledgment of the importance of our study and the need for application of machine learning methods to adolescent reproductive health using DHS data from 12 East African countries is appreciated. We acknowledge your concerns related to scientific clarity, transparency, and presentation, and we have undertaken major revisions to address these issues throughout the manuscript. In particular, we have made clear the methodological rationale, improved the explanation of our analytic approach, improved the organization of the text, and made sure that relevant details are presented clearly within the text. We hope our revision helps meet your expectations and improves the overall quality of the study.

1. Scientific Content and Justification
• You have applied machine learning (ML) to classify knowledge of the fertility period (KFP). However, you have not clearly explained why ML is needed over traditional statistical methods.
• Please explain how ML improves prediction or policy insights in this context. You may consider comparing with logistic regression outputs as a baseline.

**Author response**: Thank you for your insightful comment. In response, we have revised the manuscript to better explain the rationale for using machine learning over traditional statistical methods. We now explain how ML techniques offer improved predictive performance and flexibility in handling complex nonlinear relationships among variables. Additionally, we included a comparison with logistic regression outputs to provide a baseline and demonstrate the added value of ML in this context.

2. Outcome Variable and Definitions
• You have defined "accurate knowledge" of the fertility period as only the response “middle of the cycle.” This is a narrow definition. Please justify this approach with references. Consider whether other responses could be partially correct.

**Author response:** Thank you for your thoutghful coments. We have amended the manuscript by justifying the meaning of accurate knowledge of fertility period and inaccurate knowledge of fertility period. We have now included this clarification and supporting references in the revised Methods section to enhance transparency and strengthen the validity of our outcome variable.

3. Methodology and Model Transparency
   • You have used 10 ML algorithms and SMOTE for class balancing. While this is good, the reason for choosing SMOTE over other techniques is not clearly explained.
   • You should provide more details on:
   o Feature selection steps (Boruta output)
   o Data preprocessing
   o Handling of missing data
   o Hyperparameter tuning: why did default settings perform better than grid search or Optuna?

**Author response:** We appreciate your clear and comprehensive review. We have answered all your comments in the revised manuscript by including the relevant data. In particular, we clarified the reasons for choosing SMOTE resampling technique to balance the classes, explained our feature selection approach using Boruta, discussed our methodology for preprocessing data and managing missing data, and mentioned our reflection on hyperparameter tuning, including why default settings did better than grid search and Optuna in this case. We hope these additions improve the transparency of our methods and the overall clarity of the paper.

4. Performance Metrics
• You have reported accuracy and AUC but not F1-score, confusion matrix, or precision-recall. These are important, especially with imbalanced data.
• Please include confidence intervals for AUC and other metrics.

**Author response:** Thank you for your valuable comment regarding performance metrics and confidence interval. For this study we chose to report sensitivity, specificity, positive predictive value (PPV), negative predictive value (NPV), accuracy, and AUC as our primary performance metrics, as they are familiar to public health and clinical research and provide alternative and interpretable insights for evaluating imbalanced data. These metrics, specifically sensitivity and specificity, are especially relevant for understanding how the model performed in identifying adolescents with good fertility knowledge and for allowing us to undertake targeted interventions. PPV and NPV are particularly helpful in population applications, as they capture relative prevalence and help guide resource allocation. While we acknowledge the value of F1 score, precision, and recall in machine learning frameworks, they are more threshold-dependent and less intuitive for stakeholders in health education; we found the selected metrics more aligned with the goals of this study. Regarding confidence intervals, we did not include them. Our primary focus was on model development, feature selection, and comparative performance using cross-validation, where point estimates are commonly used and statistical inference is not strictly required.

5. Interpretation of SHAP and Findings
• SHAP plots are a strength, but their interpretation is not clear. Please explain what the plots mean in simple terms and relate them to the real-world context.
• You list top features (e.g., education, wealth, country) but do not explain how these insights can help program planners or health educators.

**Author response:** We appreciate you bringing attention to the importance of the SHAP plots and your constructive feedback. In our revision, we simplified the description of the SHAP plots and their practical application in the real world. We have included and explained how the identified top features help program planners or health educators.  We have added the interpretation in the Results and Discussion sections to make the findings more accessible and actionable for both scientific and policy audiences.

6. Language and Grammar
   • The manuscript contains many grammatical errors and awkward sentences. Examples include:
   o "Random forest was emerged..." → should be "Random forest emerged..."
   o "Bees warm plot" → should be "beeswarm plot"

• Please revise the entire manuscript carefully for grammar and clarity. A professional language edit is recommended.

**Author response:** Thank you in advance for pointing out the language and grammar issues. We have carefully reviewed the manuscript and have corrected the grammatical mistakes, poor word choice, and unclear terminology, including the ones you mentioned. The revised version has been wholly edited to provide improved clarity, readability, and proper scientific expression

7. Ethics and Data Availability
   • You have clearly stated that DHS data was used and ethical clearance obtained. This is satisfactory.
   • However, you should consider sharing your code or scripts (e.g., in a GitHub link) to improve transparency and reproducibility.

**Author response:** Thank you for your considerate suggestion. We entirely agree that the sharing of code would promote transparency and reproducibility. For now, we have chosen not to share our scripts publicly as we have an additional internal policy on data sharing and because we continue to develop possible resulting analyses. We have ensured that all methodological steps are clearly described in the manuscript to support reproducibility, and we remain open to sharing additional details upon reasonable request.

8. Conclusion and Implications
   • Your conclusion repeats the results but does not offer clear recommendations for policy, programs, or further research.
   • Please state how governments or NGOs can use your findings, especially in adolescent reproductive health programs.
   • Some tables are too long and difficult to read.
   • Please format tables clearly and ensure figure legends are complete and explained in the text.

**Author response:** We appreciate your constructive feedback. We have made edits to the Conclusion section to not only summarize the results, but also state clear recommendations for policy, programs and future research. More specifically, we clearly highlight how governments and NGOs can utilize our findings to inform targeted adolescent reproductive health interventions. Additionally, we have reformatted lengthy tables for improved readability and ensured that all figure legends are complete and clearly explained in the text.

**With regards!**

**Thank you!**

**Yours sincerely**

**Andualem Addisu Birlie (Correspondence author)**
